# Comparative analysis of the biological characteristics and mechanisms of azole resistance of clinical *Aspergillus fumigatus* strains

**DOI:** 10.3389/fmicb.2023.1253197

**Published:** 2023-11-09

**Authors:** Meng Zeng, Xue Zhou, Chunhong Yang, Yanfei Liu, Jinping Zhang, Caiyan Xin, Gang Qin, Fangyan Liu, Zhangyong Song

**Affiliations:** ^1^School of Basic Medical Sciences, Southwest Medical University, Luzhou, China; ^2^Department of Clinical Laboratory, Yongchuan Hospital of Chongqing Medical University, Chongqing, China; ^3^Department of Clinical Laboratory, The Affiliated Hospital of Qingdao University, Qingdao, China; ^4^Department of Otolaryngology Head and Neck Surgery, The Affiliated Hospital of Southwest Medical University, Luzhou, China

**Keywords:** biological characteristics, azole resistance, virulence, *cyp51A*, *Aspergillus fumigatus*

## Abstract

*Aspergillus fumigatus* is a common causative pathogen of aspergillosis. At present, triazole resistance of *A. fumigatus* poses an important challenge to human health globally. In this study, the biological characteristics and mechanisms of azole resistance of five *A. fumigatus* strains (AF1, AF2, AF4, AF5, and AF8) were explored. There were notable differences in the sporulation and biofilm formation abilities of the five test strains as compared to the standard strain AF293. The ability of strain AF1 to avoid phagocytosis by MH-S cells was significantly decreased as compared to strain AF293, while that of strains AF2, AF4, and AF5 were significantly increased. Fungal burden analysis with *Galleria mellonella* larvae revealed differences in pathogenicity among the five strains. Moreover, the broth microdilution and *E*-test assays confirmed that strains AF1 and AF2 were resistant to itraconazole and isaconazole, while strains AF4, AF5, and AF8 were resistant to voriconazole and isaconazole. Strains AF1 and AF2 carried the *cyp51A* mutations TR34/L98H/V242I/S297T/F495I combined with the *hmg1* mutation S541G, whereas strains AF4 and AF8 carried the *cyp51A* mutation TR46/Y121F/V242I/T289A, while strain AF5 had no *cyp51A* mutation. Real-time quantitative polymerase chain reaction (RT-qPCR) analysis revealed differences in the expression levels of genes associated with ergosterol synthesis and efflux pumps among the five strains. In addition, transcriptomics, RT-qPCR, and the NAD^+^/NADH ratio demonstrated that the mechanism of voriconazole resistance of strain AF5 was related to overexpression of genes associated with energy production and efflux pumps. These findings will help to further elucidate the triazole resistance mechanism in *A. fumigatus.*

## Introduction

*Aspergillus fumigatus* is a saprophytic fungus that widely exists in hospitals, gardens, and fields (Burks et al., 2021; Zhou et al., 2022), and the most common filamentous pathogenic fungus encountered in clinical practice, followed by *A. flavus*, *A. niger*, and *A. terreus* (Cadena et al., 2021). Aspergillosis is a spectrum of infections typically caused by *A. fumigatus*, which is traditionally classified as allergic bronchitis pulmonary aspergillosis (ABPA) (Eraso et al., 2020), chronic pulmonary aspergillosis (CPA) (Sehgal et al., 2020), and invasive aspergillosis (IA) (Snelders et al., 2011). ABPA is a hypersensitive lung disease that mainly occurs in patients with asthma and cystic fibrosis (El-Baba et al., 2020). CPA usually occurs in immunocompetent individuals with lung diseases, such as cavitary pulmonary tuberculosis, chronic obstructive pulmonary disease, and sarcoidosis (Smith and Denning, 2011). Pulmonary aspergillus disease is commonly encountered in clinical practice. IA is the most severe form of pulmonary aspergillus with a mortality rate exceeding 50% (Brown et al., 2012). Recent global estimates have shown that aspergillosis accounts for about 5 million cases of ABPA (Agarwal et al., 2020), 3 million cases of CPA, and one-quarter million cases of IA annually (Bongomin et al., 2017).

At present, antifungal drugs for the treatment of aspergillosis mainly include azoles, echinocandins, and polyenes (Galiger et al., 2013). Azoles, the most commonly used first-line drugs against aspergillosis, include isavuconazole (ISA), itraconazole (ITR), posaconazole (POS), and voriconazole (VRC). Cyp51 (cytochrome P450 family 51 subfamily A member 1) is an enzyme encoded by *cyp51A* and *cyp51B*, which plays an important role in sterol biosynthesis. By binding with Cyp51, azoles inhibit the synthesis of ergosterol on the fungal cell membrane, thereby inhibiting synthesis of sterol 14α-demethylase as an antifungal effect (Mellado et al., 2001; Parker et al., 2014; Peyton et al., 2015). At present, the high death rate of aspergillosis is mainly due to the limited choice of antifungal drugs and the increasing rate of resistance of clinical *A. fumigatus* strains. With the exception of Antarctica, azole-resistant *A. fumigatus* has been reported worldwide (Burks et al., 2021). According to data released from Netherlands, the prevalence of azole-resistant *A. fumigatus* increased from 0.79% in 1996–2001 to 11.30% in 2013–2018 (Buil et al., 2019; Lestrade et al., 2020). Recently, 21 research centers worldwide reported that the incidence of triazole-resistant *A. fumigatus* ranged from 3.2 to 26.1% (van der Linden et al., 2015), while the prevalence of *Aspergillus* is increasing in some parts of the world (Yang et al., 2021). The increased prevalence of drug-resistant *A. fumigatus* has led to increased treatment failure and healthcare costs, thus, emerging as public health concern globally. An insertion mutation of a tandem repeat sequence in the promoter region of *cyp51A* was confirmed to change to the amino acid coding sequence resulting in resistance to azoles (Snelders et al., 2010). The resulting mutations mainly include TR34/L98, TR46/Y121F/T289A, TR53, and TR120 (Alvarez-Moreno et al., 2017; Hare et al., 2019). Of these mutations, TR34/L98 and TR46/Y121F/T289A are the most common in clinical strains. Additional mutations to the Cyp51A amino acid coding sites include G54, Y121, G138, P216, F219, M220, A284, Y431, G432, G434, and G448 (Pérez-Cantero et al., 2020). Besides changes to the amino acid sequence, overexpression of genes associated with drug efflux pumps has been implicated in azole resistance of *A. fumigatus*. The adenosine triphosphate (ATP)-binding cassette (ABC) efflux pump and major facilitator superfamily (MFS) transporters play key roles in drug efflux. A recent investigation found that the *A. fumigatus* genome has 49 and 278 genes coding for proteins that comprise ABC efflux pumps and MFS transporters, respectively (Arastehfar et al., 2021). However, relatively few genes coding for drug efflux pumps have been confirmed, as most other members are redundant. Nonetheless, the mechanisms underlying *A. fumigatus* resistance to azoles remain unclear.

In this study, five strains of *A. fumigatus* (AF1, AF2, AF4, AF5, and AF8) were identified by morphological and molecular analyses. In addition, the biological characteristics of sporulation, biofilm formation, evasion of phagocytosis, virulence, and drug sensitivity of the five strains were investigated. Sanger sequencing, three-dimensional (3D) protein construction, high-performance liquid chromatography (HPLC), and real-time quantitative polymerase chain reaction (RT-qPCR) were used to clarify the mechanisms underlying drug resistance of the five strains. Furthermore, transcriptomics, RT-qPCR, and biochemical analyses were employed to explore the specific drug resistance mechanism of the AF5 strain.

## Materials and methods

### Strains and cell line

In total, 45 *A. fumigatus* strains from different clinical specimens were collected from May 2019 to April 2021. The patient information related to these strains in the study was showed in Supplementary Table 1. The AF293 strain was used as a control. All strains were stored in sterile ddH_2_O at room temperature (Hartung de Capriles et al., 1989). Prior to analysis, the strains were cultured on PDA at 37°C for 3–7 days. Mouse alveolar macrophages (MH-S cells) were donated by Professor Jia Jing of the Affiliated Hospital of Southwest Medical University (Luzhou, China).

### Identification of strains and spot assay

The AF1, AF2, AF4, AF5, AF8, and AF293 strains were cultured on PDA at 37°C for 5 days. Colonies were selected and stained with lactophenol cotton blue stain solution. Morphological characteristics of conidia, conidiophores, and mycelia were observed under a microscope.

Each strain was cultured in Roswell Park Memorial Institute (RPMI) 1640 liquid medium (Cytiva, Marlborough, MA, USA) at 37°C for 48 h. Afterward, the mycelia were collected. Genomic DNA was extracted using a commercial DNA extraction kit (Sangon Biotech Co. Ltd., Shanghai, China) in accordance with the manufacturer’s instructions. The internal transcribed spacer (*ITS*) sequences of the test strains were amplified by RT-qPCR with the primers listed in Supplementary Table 2. The Sanger sequencing results were compared with the Basic Local Alignment Search Tool.^1^ An evolutionary tree of the *ITS* sequences was generated with Molecular Evolutionary Genetics Analysis software.^2^

Conidia of *A. fumigatus*, cultured for 5 days, were eluted with 0.05% Tween 80 and diluted to 1 × 10^6^ cells/mL. Then, 3 μL of diluted conidia were spotted in the center of PDA plates (diameter, 9 cm) and incubated at 37°C for 36 and 120 h, respectively. Then, all conidia were eluted with 0.05% Tween 80 in sterile water and counted under a light microscope (magnification, 400 ×).

### Observation and detection of biofilm formation

The biofilm formation assay was performed as described in a previous report (Zhang et al., 2022). Briefly, 2 mL of conidia (1.0 × 10^5^ cells/mL) were added to the wells of a 12-well cell culture plate, covered with round coverslips, and cultured at 37°C for 24 h. Then, the unattached spores and mycelia were washed three times with PBS. Afterward, 1 mL of calcofluor white stain (CFW) (Beijing Solarbio Science & Technology Co., Ltd.) and 20 μL of 10% KOH were added to each well and the plate was incubated at room temperature for 3–5 min. Biofilm formation was observed with a confocal laser scanning microscope (Olympus Corporation, Tokyo, Japan).

To further confirm the biofilm formation ability of the test strains, 2 mL of spore suspensions (1.0 × 10^5^ cells/mL) were added into the wells of 12-well cell culture plates and cultured at 37°C for 24 h. Afterward, the unattached spores and mycelia were washed three times with PBS. Then, 200 μL of a mixture of 2,3-bis(2-methoxy-4-nitro-5-sulfophenyl)-5-[(phenylamino)-carbonyl]-2H-tetrazolium hydroxide sodium salt and menadione were added to each well and the plate was incubated at 37°C for 2 h in the dark. Finally, the supernatant was transferred to the wells of a new 96-well plate and absorbance was measured at 490 nm.

### Phagocytosis assay

The phagocytosis assay was performed as previously described (Lim et al., 2022). Briefly, MH-S cells were cultured in complete Dulbecco’s modified Eagle’s medium (Thermo Fisher Scientific, Waltham, MA, USA) containing 10% fetal bovine serum and 1% penicillin-streptomycin. Then, 1 mL of the cultured MH-S cells (1 × 10^5^ cells/mL) was added to each well of a 12-well plate, covered with round coverslips, and cultured overnight at 37°C under an atmosphere of 5% CO_2_/95% air. Afterward, 1 mL of conidia (1 × 10^6^ cells/mL) was added to each well and the plate was cultured for 2 h under an atmosphere of 5% CO_2_/95% air. The 12-well plate was washed three times with PBS pre-cooled at 4°C. Then, the round coverslips were stained with 50 μg/mL of CFW and observed under a microscope. The phagocytosis percentage (Eq. 1) and phagocytosis index (Eq. 2) were calculated in reference to the microscope images and repeated independently three times. In total, 200 MH-S cells were randomly counted.

Phagocytosis rate = number of MH-S cells of phagocytizing conidia/200 × 100% (Eq. 1).

Phagocytosis index = total number of phagocytic conidia/number of MH-S cells of phagocytizing conidia (Eq. 2).

### Infection of the *Galleria mellonella*

*G. mellonella* larvae (*n* = 10/group) were injected with 10 μL of the test strain in suspension through the last pro-leg into the hemocoel using a 50-μL microinjector (Shanghai Gaoge Industry and Trade Co., Ltd., Shanghai, China) (Özkan and Coutts, 2015). Untreated, pierced only, and PBS-injected groups were used as controls. The experimental groups were injected with 10 μL of conidia (1 × 10^7^ cells/mL). After injection, each group of larvae was divided into two petri dishes and cultured at 37°C in the dark. The larvae were photographed and morphological changes were recorded every 24 h. Each experiment was repeated three times.

Different groups of infected larvae were sacrificed at 24 or 72 h after infection. The whole larvae were macerated in microtubes with 1 mL of ddH_2_O. Afterward, 50 μL of the dilution were seeded on PDA medium and the plates were incubated at 37°C. Each experiment was repeated three times.

### Antifungal susceptibility testing

Drug sensitivity of the five strains was determined in accordance with Clinical and Laboratory Standards Institute (CLSI) document M38-A2—Reference Method for Broth Dilution Antifungal Susceptibility Testing of Filamentous Fungi (Wayne, 2008). The antifungal agents were used at the following concentrations: fluconazole (FLC), 64–0.0625 μg/mL; anidulafungin (ANI), amphotericin B (AmB), caspofungin (CAS), ISA, micafungin (MF), POS, and VRC, 32–0.03125 μg/mL. The conidia (1.0 × 10^5^ cells/mL) were dispensed into triplicate wells of 96-well microtiter plates and incubated at 37°C for 48 h. Conidia in drug-free wells were used as growth controls. The minimum inhibitory concentration (MIC) was defined as the lowest drug concentrations that caused complete visible inhibition of growth after incubation at 37°C for 48 h. The minimum effective concentration (MEC) was defined as the lowest drug concentration causing the mycelia to become shorter and thicker, as observed under a microscope, after incubation at 37°C for 48 h (Subcommittee on Antifungal Susceptibility Testing of the ESCMID European Committee for Antimicrobial Susceptibility Testing, 2008).

For the strip tests, the conidia (1.0 × 10^6^ cells/mL) were evenly coated on RPMI 1640 solid medium using cotton swabs. Once the plate was dry, an *E*-test strip (Liofilchem s.r.l., Roseto degli Abruzzi, Italy) was attached to the center of the medium with sterile tweezers. The results were read after incubation at 37°C for 24 or 48 h. The MIC was determined in reference to the scale at the intersection of the inhibition zone and the *E*-test strip. For both methods, strain AF293 was used for quality control. Each experiment was repeated three times.

### Sequencing of *cyp51A*, *cyp51B*, and *hmg1*

Sanger sequencing of *cyp51A*, *cyp51B*, and *hmg1*, in addition to the promoter region of *cyp51A*, was conducted. The sequences were compared using DNAMAN software (Lynnon Biosoft, San Ramon, CA, USA) to identify short tandem repeats and amino acid mutations. The 3D structures of the Cyp51A proteins of the test strains were generated and analyzed with Discovery Studio software (BIOVIA, San Diego, CA, USA).

### Analysis of ergosterol content

The ergosterol content was determined by HPLC (1260 infinity II; Agilent Technologies, Inc., Santa Clara, CA, USA) as described in a previous report (Zhang et al., 2022). Briefly, conidia (1 × 10^6^ cells/mL) were inoculated into 50 mL of RPMI 1640 medium and cultured at 37°C for 48 h at 200 rpm. The samples were exposed to ultrasonic waves at 40 kHz. Following the addition of methanol (w/w), the supernatant was collected by centrifugation as the sample to be tested. An ergosterol standard (Shanghai Macklin Biochemical Co., Ltd., Shanghai, China) was used as a reference and 100% methanol as a negative control. The concentration of ergosterol = (peak area of experimental group × concentration of ergosterol standard group)/peak area of ergosterol standard product. Each experiment was repeated three times.

### RT-qPCR and transcriptomic analysis

Conidia (1.0 × 10^6^ cells/mL) of the test strain were inoculated into RPMI 1640 medium and cultured at 37°C for 16 h at 200 rpm, and then co-cultured with or without drugs for 0, 4, 8, or 12 h. The mycelia were collected and frozen in liquid nitrogen until assayed. Total RNA extraction, reverse transcription, and RT-qPCR reactions were performed using commercial kits (Takara Bio, Inc., Shiga, Japan) in accordance with the manufacturer’s instructions. Transcript levels were determined by a ABI 7500Fast Real-Time PCR Detection System (Thermo Fisher Scientific, Waltham, MA, USA) and measured with the 2^–ΔΔCT^ method against β*-tubulin* as an internal control (Vandesompele et al., 2002).

To further clarify the azole resistance mechanism, conidia of strain AF5 prepared in RPMI 1640 medium were treated with or without VRC (MIC = 0.5) and incubated at 37°C for 8 h. Total RNA was collected and sequenced by Biomarker Technologies (Qingdao, China) with an Illumina RNA sequencing system (Illumina, Inc., San Diego, CA, USA). Then, quality control detection of the raw sequence data was performed on the Biomarker platform.^3^ Sequencing alignment was performed using the Hisat2 (version 2.2.1, and the reference genome is *Aspergillus_fumigatus*.GCF_000002655.1_ASM265v1.genome.fa). After that the samtools (version 1.9) was used to sort the sam files to bam files, and then matched Reads were assembled and quantified using the StringTie tool (version 1.3.4d). The tool for differentially expressed genes (DEGs) analysis is DESeq2 (version 1.6.3). Further gene function annotation was included COG (Cluster of Orthologous Groups of proteins), KO (Kyoto Encyclopedia of Genes and Genomes (KEGG) Ortholog database), and GO (Gene Ontology). The time-specific expression patterns of genes were determined by RT-qPCR analysis. Raw sequence data were deposited in the Genome Sequence Archive of the Beijing Institute of Genomics (Chinese Academy of Sciences, Beijing, China; accession number CRA011198).

### Determination of nicotinamide adenine dinucleotide (NAD^+^) and NADH contents

The contents of NAD^+^ and NADH were measured using a commercial kit (Beyotime Institute of Biotechnology, Shanghai, China) as described in a previous report (Hsieh et al., 2020). Briefly, the conidia (1.0 × 10^6^ cells/mL) of strain AF5 were inoculated into RPMI 1640 medium and cultured at 37°C for 16 h, and then co-cultured with VRC (MIC = 0.5) for an additional 0, 4, 8, or 12 h. Afterward, the mycelia were collected and frozen in liquid nitrogen until assayed. Following the addition of working solution, the supernatant of each sample was collected and divided into two aliquots. One aliquot was directly transferred to the wells of a 96-well plate and the other was incubated at 60°C for 30 min to decompose NAD^+^ into NADH, and then transferred to the wells of a 96-well plate to determine the intracellular content of NADH.

### Statistical analysis

Data analyses were conducted using GraphPad Prism software v9.0 (GraphPad Software, Inc., San Diego, CA, USA). Group comparisons were performed with the Student’s *t*-test or one-way analysis of variance. Mortality curves were generated by the Kaplan–Meier method and compared with the log-rank test. Each experiment was repeated three times. A probability (*p*) value < 0.05 was considered statistically significant.

## Results

### Identification of strains and comparisons of sporulation ability

**FIGURE 1:**
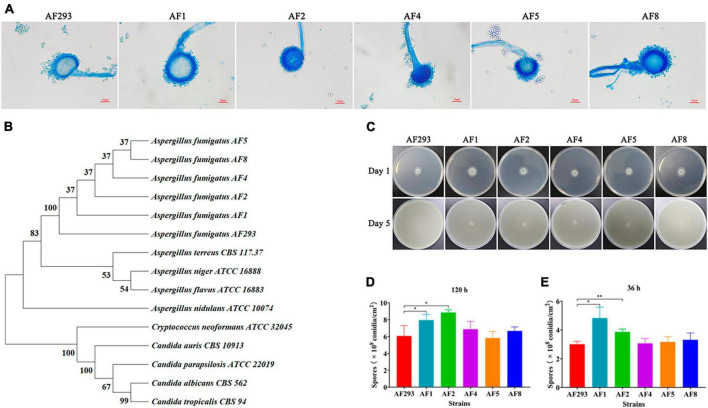
Identification of isolates and analysis of sporulation ability. **(A)** Morphological observation of conidiophores (magnification, 40 × ). Scale bar = 10 μm. **(B)** Phylogenetic analysis of ITS sequences. **(C)** Observations of colony color and morphology. Test strains were cultured on PDA medium at 37°C. **(D,E)** Sporulation statistics at 36 and 120 h. *p < 0.05 and **p < 0.01 vs. strain AF293.

The conidiophores of *Aspergillus* strains AF1, AF2, AF4, AF5, and AF8 were stained with lactophenol cotton blue stain solution and observed under a microscope (Figure 1A). Phylogenetic tree analysis of the *ITS* sequences revealed that all five test strains (AF1, AF2, AF4, AF5, and AF8) were clustered with strain AF293 (Figure 1B), confirming that all five test strains were *A. fumigatus*.

All five test strains were cultured on PDA medium for 5 days. As compared to strain AF293, there were no significant differences in the colony color and morphology of the five test strains (Figure 1C). As compared to strain AF293, the conidia yields of strains AF1 and AF2 were significantly increased by incubation for 36 and 120 h, while there was no significant difference among strains AF4, AF5, and AF8 (Figures 1D, E).

### Differences in biofilm formation and evasion of phagocytosis among the test strains

**FIGURE 2:**
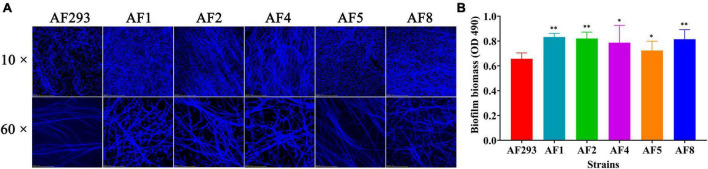
Biofilm formation ability of the test strains. The test strains were cultured at 37°C for 24 h. **(A)** Biofilm formation of the test strains after staining with CFW was observed with a confocal laser scanning microscope. Scale bar = 100 μm (magnification, 10 × ) or 20 μm (magnification, 60 × ). **(B)** Quantification of biofilm formation of the test strains using the 2,3-bis(2-methoxy-4-nitro-5-sulfophenyl)-5-[(phenylamino)-carbonyl]-2H- tetrazolium hydroxide sodium salt assay. *p < 0.05 and **p < 0.01 vs. strain AF293.

To explore the differences biofilm formation ability among test strains, as compared to strain AF293, strains AF1, AF2, AF4, AF5, and AF8 formed more and denser biofilms (Figure 2A), as confirmed by significantly increased optical density at 490 nm (Figure 2B).

**FIGURE 3:**
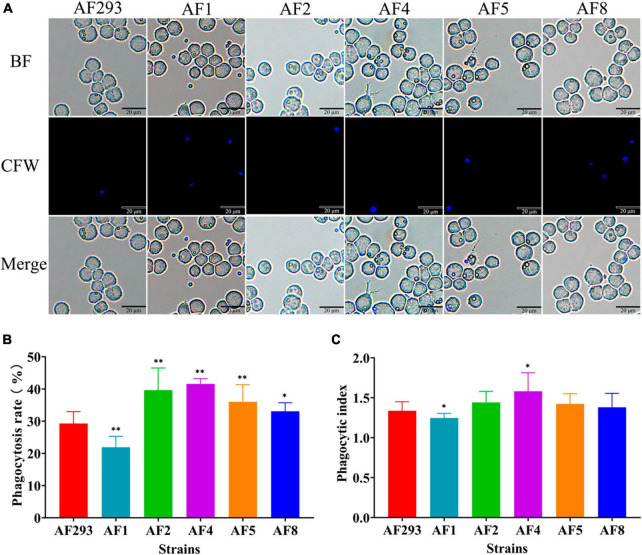
Comparative analysis of anti-phagocytosis ability of the test strains. MH-S cells were challenged with fivefold *A. fumigatus* conidia and then incubated for at 37°C for 2 h under an atmosphere of 5% CO_2_/95% air. **(A)** Phagocytosis to *A. fumigatus* conidia by MH-S cells was observed with a fluorescence microscope. Scale bar = 20 μm. **(B)** Statistical analysis of the phagocytosis rate. **(C)** Statistical analysis of the phagocytic index. *p < 0.05 and **p < 0.01 vs. strain AF293.

To explore the interaction between the *A. fumigatus* conidia and MH-S cells, the conidia of the test strains were phagocytosed by MH-S cells after co-culture for 2 h, as observed with a fluorescence microscope (Figure 3A). As compared to strain AF293, phagocytosis of the conidia of strains AF2, AF4, AF5, and AF8 was significantly increased, while that of strain AF1 conidia was significantly decreased (Figure 3B). Further phagocytic index analysis, representing the average number of conidia phagocytosed per MH-S cell, showed that the phagocytosis index of MH-S cells to strain AF1 was significantly decreased, while that of strain AF4 was significantly increased (Figure 3C). These results indicate enhanced ability of strain AF1 to avoid phagocytosis, while the ability of the other strains to avoid phagocytosis was decreased.

### Differences in virulence among the test strains

**FIGURE 4:**
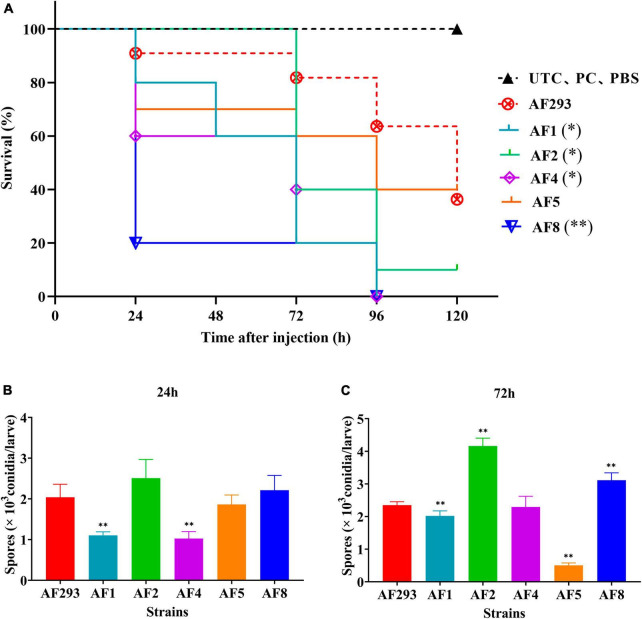
Survival and fungal burden analysis of *G. mellonella* larvae. **(A)** Survival curves of the model groups injected with *A. fumigatus* conidia. UTC: blank control group, PC: puncture without injection group, PBS: PBS injection group. Fungal burden of *G. mellonella* larvae at 24 h **(B)** and 72 h **(C)** after infection. *p < 0.05 and **p < 0.01 vs. strain AF293.

The *G. mellonella* larvae infection models were subsequently opted to explore variations in virulence among test strains. *G. mellonella* larvae infected with *A. fumigatus* conidia begin to melanize and die after 24 h. However, there was no significant correlation between melanization and strains (Supplementary Figure 1). As compared to strain AF293, the larvae survival rate was significantly decreased in the AF1, AF2, AF4, and AF8 model groups at 120 h after inoculation with 10^7^ cells/mL (Figure 4A). The fungal burden was significantly decreased in the AF1 and AF4 groups at 24 h (Figure 4B). At 72 h, the fungal burden was significantly decreased in the AF1 and AF5 groups, while significantly increased in the AF2 and AF8 groups (Figure 4C).

### Susceptibility to AmB, echinocandins, and triazoles

The MIC_90_ and MEC of azoles, AmB, and polyenes against the test strains are shown in Table 1. All test strains were resistant to FLC, as determined by the broth microdilution method, while strains AF1 and AF2 were resistant to ITR and ISA, and sensitive to AmB, ANI, CAS, MF, POS, and VRC. Meanwhile, strains AF4, AF5, and AF8 were resistant to VRC and ISA, and sensitive to AmB, ANI, CAS, MF, ITR, and POS. In addition, the *E*-test results were consistent with the results of the broth microdilution method (Supplementary Figure 2).

**TABLE 1:** Sensitivity of antifungal drugs of test strains.

Strains	MIC_90_ (μg/mL)					MEC (μg/mL)		
	**FLC**	**ITR**	**ISA**	**VRC**	**AmB**	**ANI**	**CAS**	**MF**
AF1	>64	>32	8	0.25	0.25	<0.0625	0.125	<0.0625
AF2	>64	>32	16	0.5	<0.25	0.0625	0.125	0.0625
AF4	>64	2	16	32	0.5	<0.0625	0.125	<0.0625
AF5	>64	2	>16	16	0.5	<0.0625	0.25	0.25
AF8	>64	2	>16	32	0.5	<0.0625	<0.0625	<0.0625
AF293	>64	0.25	0.125	0.125	0.25	0.0625	0.125	0.0625

ANI, anidulafungin; AmB, amphotericin B; CAS, caspofungin; FLC, fluconazole; ITR, itraconazole; ISA, isavuconazole; MF, micafungin; VRC, voriconazole; MIC_90_, minimal drug concentration that inhibits the growth of 90%; MEC, minimum effective concentration.

### *cyp51A*-mediated mechanism of azoles resistance

Sanger sequencing and comparison analysis showed that strains AF1 and AF2 carried the *cyp51A* mutations TR34/L98H/S297T/F495I, strains AF4 and AF8 carried the *cyp51A* mutations TR46/Y121F/T289A, and strain AF5 carried no mutation (data not shown). Further analysis with Discovery Studio software showed that there was no difference in the 3D structure of Cyp51A between strains AF293 and AF5. Moreover, although there were small spatial differences in the branch chain of Cyp51A among the other four strains, the spatial structure of the main chain conformation was consistent with that of strain AF293 (Supplementary Figure 3). HPLC analysis found that there was no significant difference in ergosterol content (data not shown). In addition, Sanger sequencing and comparison analysis showed that none of the five test strains carried a *cyp51B* mutation, while strains AF1 and AF2 carried the *hmg1* mutation S541G (data not shown).

**FIGURE 5:**
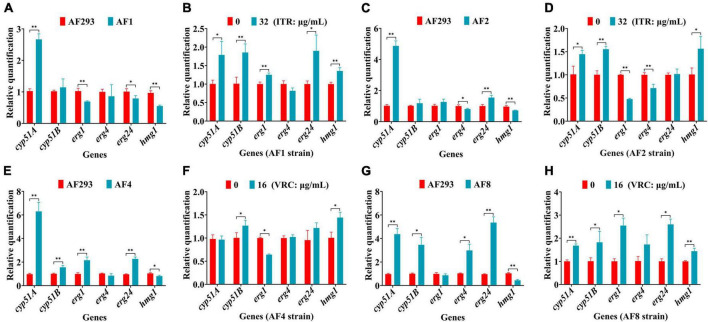
Real-time quantitative polymerase chain reaction (RT-qPCR) analysis of genes associated with ergosterol synthesis. After incubation at 37°C for 16 h, the culture was continued for another 8 h with or without the addition of ITR or VRC. **(A,C,E,G)** Changes in gene expression levels of the four strains. **(B,D,F,H)** Gene expression levels following administration of ITR (32 μg/mL) or VRC (16 μg/mL) (MIC = 0.5). *p < 0.05 and **p < 0.01 vs. untreated strain AF293.

Differences in the expression levels of genes associated with ergosterol synthesis, including *cyp51A*, *cyp51B*, *erg1*, *erg4*, *erg24*, and *hmg1*, were detected by RT-qPCR analysis. As compared to strain AF293, *cyp51A* expression was significantly up-regulated, while the expression levels of *erg1*, *erg24*, and *hmg1* genes were significantly down-regulated in strain AF1 (Figure 5A). Treatment of strain AF1 with ITR (32 μg/mL for 8 h) significantly up-regulated the expression levels of *cyp51A*, *cyp51B*, *erg1*, *erg24*, and *hmg1* (Figure 5B). As compared to strain AF293, *cyp51A* and *erg24* were significantly up-regulated, while *erg4* and *hmg1* were significantly down-regulated in strain AF2 (Figure 5C). Treatment of strain AF2 with ITR (32 μg/mL for 8 h) significantly up-regulated *hmg1* and significantly down-regulated *cyp51A* and *cyp51B* (Figure 5D). As compared to strain AF293, *cyp51A*, *cyp51B*, *erg1*, and *erg24* were significantly up-regulated, while *hmg1* was significantly down-regulated in strain AF4 (Figure 5E). Treatment of strain AF4 with VRC (16 μg/mL for 8 h) significantly up-regulated *cyp51A* and *hmg1*, and significantly down-regulated *erg1* (Figure 5F). As compared to strain AF293, *cyp51A*, *cyp51B*, *erg4*, and *erg24* were significantly up-regulated, while *hmg1* was significantly down-regulated in strain AF8 (Figure 5G). Treatment of strain AF8 with VRC (16 μg/mL for 8 h) significantly up-regulated *cyp51A*, *cyp51B*, *erg1*, *erg24*, and *hmg1* (Figure 5H). These results indicate that the resistance mechanism of strains AF1, AF2, AF4, and AF8 is related to *cyp51* mutation or overexpression.

### Non-*cyp51A*-mediated mechanism of azole resistance

**FIGURE 6:**
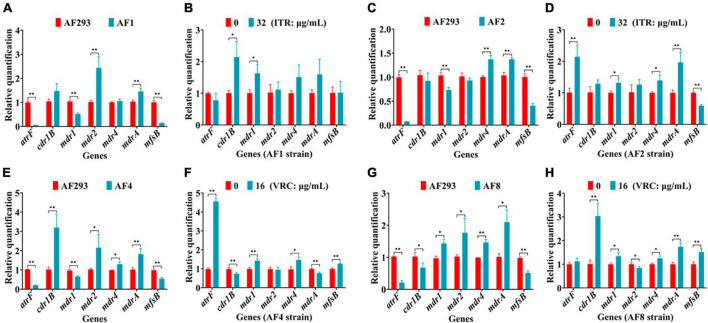
Real-time quantitative polymerase chain reaction (RT-qPCR) analysis of genes associated with drug efflux pumps. After incubation at 37°C for 16 h, the culture was continued for another 8 h with or without administration of ITR or VRC. **(A,C,E,G)** Changes to gene expression levels of the four strains. **(B,D,F,H)** Gene expression levels following administration of ITR (32 μg/mL) or VRC (16 μg/mL) (MIC = 0.5). *p < 0.05 and **p < 0.01 vs. untreated strain AF293.

Changes to the expression levels of genes associated with drug efflux pumps (e.g., *atrF*, *cdr1B*, *mdr1*, *mdr2*, *mdr4*, *mdrA*, and *mfsB*) were further confirmed. As compared to strain AF293, *mdr2*, and *mdrA* were significantly up-regulated, while *atrF*, *mdr1*, and *mfsB* were significantly down-regulated in strain AF1 (Figure 6A). Treatment of strain AF1 with ITR (32 μg/mL for 8 h) significantly up-regulated *cdr1B* and *mdr1* (Figure 6B). As compared to strain AF293, *mdr4* was significantly up-regulated, while *atrF*, *mdr1*, and *mfsB* were significantly down-regulated in strain AF2 (Figure 6C). Treatment of strain AF2 with ITR (32 μg/mL for 8 h) significantly up-regulated *atrF*, *mdr1*, *mdr4*, and *mdrA*, and significantly down-regulated *mfsB* (Figure 6D). As compared to strain AF293, *cdr1B*, *mdr2*, *mdr4*, and *mdrA* were significantly up-regulated, while *atrF*, *mdr1*, and *mfsB* were significantly down-regulated in strain AF4 (Figure 6E). Treatment of strain AF4 with VRC (16 μg/mL for 8 h) significantly up-regulated *atrF*, *mdr1*, *mdr4*, and *mfsB*, and significantly down-regulated *cdr1B* and *mdrA* (Figure 6F). As compared to strain AF293, *mdr1*, *mdr2*, *mdr4*, and *mdrA* were significantly up-regulated, while *atrF*, *cdr1B*, and *mfsB* were significantly down-regulated in strain AF8 (Figure 6G). Treatment of strain AF8 with VRC (16 μg/mL for 8 h) significantly up-regulated *cdr1B*, *mdr1*, *mdr2*, *mdr4*, and *mdrA*, with no significant difference in expression of *atrF* and *mfsB* (Figure 6H). These results indicate that the azole resistance mechanisms of strains AF1, AF2, AF4, and AF8 are related to overexpression of genes associated with efflux pumps.

### The mechanism of VRC resistance in AF5 strain

**FIGURE 7:**
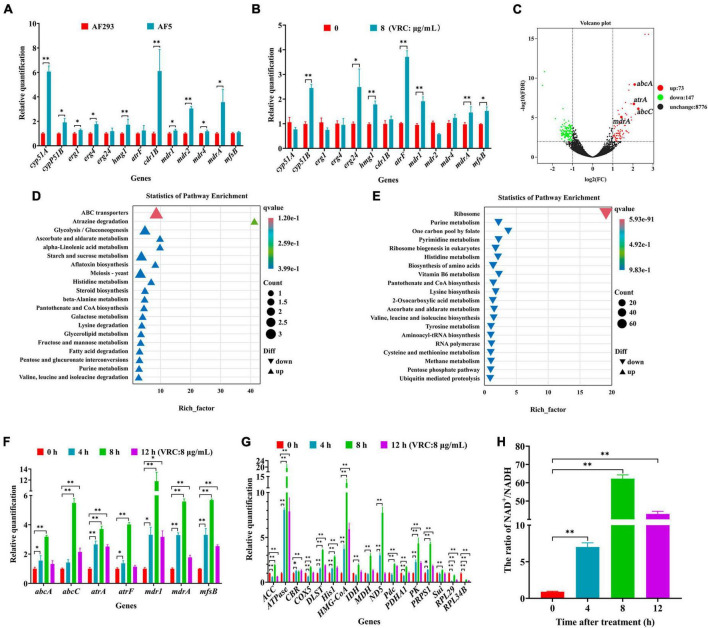
The mechanism of VRC resistance in AF5 strain. **(A)** Expression levels of genes associated with ergosterol synthesis and drug efflux pumps in strain AF5 vs. strain AF293. **(B)** Expression levels of gens associated with ergosterol syntheses and drug efflux pumps in strain AF5 treated with VRC at 8 μg/mL (MIC = 0.5). **(C)** Volcano map of differentially expressed genes. **(D,E)** Scatter plot of enriched pathways of the up- and down-regulated genes. Strain AF5 was incubated in RPIM 1640 medium for 16 h at 37°C and then treated with VRC (MIC = 0.5) for another 8 h. **(F,G)** Expression levels of gens associated with efflux pumps and energy metabolism. **(H)** Bar chart of NAD^+^/NADH. Strain AF5 was incubated in RPIM 1640 medium for 16 h at 37°C and then treated with VRC (MIC = 0.5) for another 0, 4, 8, and 12 h. *p < 0.05 and **p < 0.01 vs. 0 h.

The results of previous experiments found that *cyp51A*, *cyp51B*, and *hmg1* of strain AF5 carried no mutations, and there was no change in the ergosterol content. As compared to strain AF293, the expression levels of genes associated with ergosterol synthesis (i.e., *cyp51A*, *cyp51B*, *erg1*, *erg4*, and *hmg1*) and efflux pumps (i.e., *cdr1B*, *mdr1*, *mdr4*, and *mdrA*) were significantly up-regulated in strain AF5. After treatment with VRC (8 μg/mL for 8 h), the expression levels of genes associated with ergosterol synthesis (i.e., *cyp51B*, *erg24*, and *hmg1*) and efflux pumps (i.e., *atrF*, *mdr1*, *mdrA*, and *mfsB*) were significantly down-regulated in strain AF5 (Figures 7A, B). Transcriptomics sequencing was conducted to further explore the mechanism of VRC resistance of strain AF5. The results showed that among these DEGs, 73 genes were significantly up-regulated and 147 genes were significantly down-regulated (Figure 7C). DEGs were functionally grouped into GO classes comprising 40 functional categories (Supplementary Figure 4), and the DEGs were involved in biological processes including drug efflux, glucose metabolism, and ribosome metabolism (Supplementary Figure 5). Further analysis found that there are 32 DEGs of up-regulated genes were assigned to 20 KEGG enrichment pathways, the top one pathway is ABC efflux pumps (4.1%), and another interesting pathway is the glycolysis pathway (2.74%), whereas there are 144 DEGs of the down-regulated genes were assigned to 20 KEGG enrichment pathways, and the top one pathway is ribosome metabolic pathway (50.34%) (Figures 7D, E), which are consistent with the GO analysis.

To confirm the results of transcriptome analysis, RT-qPCR analysis of genes associated with efflux pumps and energy metabolism was performed. The results showed that the expression levels of genes associated with effluent pumps (i.e., *abcA*, *abcC*, *atrA*, *atrF*, *mdr1*, *mdrA*, and *mfsB*) were significantly up-regulated (Figure 7F), in addition to genes associated with glycolysis (i.e., *hxkA* and *PK*), the tricarboxylic acid cycle (i.e., *IDH*, *DLST*, and *MDH*), the electron transport chain (i.e., *ND5*, *CBR*, and *COX5*), the pentose phosphate pathway rate-inhibiting enzyme (i.e., *PRPS1*), the malic acid cycle (i.e., *Pdc*), and the cholesterol and fatty acid anabolic pathways (i.e., *HMG-COA* and *ACC*). Meanwhile, the expression levels of *Sui* (a key gene of ATP synthase) and ATPase-related genes were also significantly up-regulated (Figure 7G). Further biochemical analysis showed that the NAD^+^/NADH ratio was significantly increased in strain AF5 at 4, 8, and 12 h after administration of VRC (Figure 7H). These results suggest that strain AF5 responds to VRC by increasing energy production and overexpression of genes associated with efflux pumps.

## Discussion

Lactophenol cotton blue staining is a common and rapid method for identification of filamentous fungi (Thomas et al., 1991). However, the accuracy of this method for species identification is poor. Thus, molecular techniques can be added to address this shortcoming. Common genetic markers for identification of *Aspergillus* spp. include *ITS*, β*-tubulin*, *actin*, and *calmodulin* (Hong et al., 2005; Samson et al., 2007). In the present study, morphological and molecular techniques were used to assess the biological characteristics and mechanisms of azole resistance of five commonly encountered clinical *A. fumigatus* strains.

Conidia and biofilm are related to the virulence of *A. fumigatus*. The conidia of *A. fumigatus* are ubiquitous in the environment and each person inhales at least 100 conidia/day (Nywening et al., 2020). Because of the small diameter, conidia easily enter the lungs and can also be easily cleared by alveolar macrophages. However, in immunocompromised individuals, a small portion of conidia will swell, germinate, and form mycelia, which will eventually cause invasive tissue damage and lead to IA (Zhang et al., 2021). Meanwhile, the formation of biofilm by *A. fumigatus* diminishes susceptibility to antifungal drugs, thereby frequently resulting in treatment failure (Kowalski et al., 2020; Morelli et al., 2021). Biofilm is a network structure mainly composed of conidia, mycelia, and extracellular matrix. As compared to planktonic cells of the same organism, microbial cells within a biofilm are highly resistant to current antifungal drugs and have become a source of persistent infection and high pathogenicity, especially in immunocompromised patients (Beauvais and Latgé, 2015; Borghi et al., 2016; Tits et al., 2020). In this study, there were notable differences in conidia production and biofilm formation between the two azole-resistant test strains (Figures 1, 2), suggesting differences in the degrees of virulence. As a model organism, *G. mellonella* provides a reliable means to assess the virulence potential of *A. fumigatus* (Durieux et al., 2021). The process of melanization serves as a crucial indicator for *G. mellonella* in its defense against pathogens (Kavanagh and Reeves, 2004). The phagocytosis assay and *G. mellonella* infection study also confirmed differences in pathogenicity (Figures 3, 4). In addition, the prognosis of patients infected with different azole resistant strains showed that the virulence of strains on the host body was also closely related to the host’s own underlying diseases (Supplementary Table 2). However, further *in vivo* studies are needed to clarify the specific differences in the disease processes of the two azole-resistant strains.

Ergosterol stabilizes the fungal cell membrane by directly binding to phospholipids and plays important roles in cell membrane fluidity, cell cycle progression, cell morphology, and substance transport (Banerjee et al., 2014; Rana et al., 2019). Ergosterol synthesis involves the participation of many enzymes and genes (i.e., *cyp51A*, *cyp51B*, *erg1*, *erg4*, and *hmg1*) (Lan et al., 2021). Azole resistance of *A. fumigatus* is associated with a *cyp51A* mutation (Gonzalez-Lara et al., 2019), which is reported to decrease affinity of azoles and counteract the antifungal effects (van Ingen et al., 2015; Pérez-Cantero et al., 2020). Azoles target *cyp51A*, which codes for the key enzyme in ergosterol synthesis (Pontes et al., 2020). Common *cyp51A* mutations include TR34/L98, TR46/Y121F/T289A, TR53, and TR120 (Alvarez-Moreno et al., 2017; Hare et al., 2019). The results of the present study confirmed that the *cyp51A* mutations TR34/L98H/S297T/F495I conferred resistance to ITR in strains AF1 and AF2, while the *cyp51A* mutations TR46/Y121F/T289A conferred resistance to VRC in strains AF4 and AF8. In addition, Cyp51B, as a homologous protein of Cyp51A, has been confirmed in Saccharomyces cerevisiae by allogeneic expression of defective *cyp51* of *A. fumigatus*. Cyp51A and Cyp51B effectively complement each other in terms of ergosterol content and tolerance to azoles (Martel et al., 2010). In this study, there was no Cyp51B mutation to in four of the test strains. Moreover, 3-hydroxy-3-methyl-glutaryl-coenzyme A (HMG-CoA) reductase is the rate-limiting enzyme in the first step of ergosterol synthesis, thus change to the expression of the HMG-CoA-coding gene hmg1 will directly affect ergosterol synthesis (Liang et al., 2021). The different mutation sites of *hmg1* confer different patterns of resistance to triazoles by *A. fumigatus*. For example, the *hmg1* mutation S541G had no effect on azole resistance. However, the combination of *cyp51A* and *hmg1* mutations increased azole resistance (Arai et al., 2021). For example, the *hmg1* mutation S541G combined with the *cyp51A* mutation W273S or TR34/L98H leads to drug resistance (Resendiz-Sharpe et al., 2020). In the present study, the *hmg1* mutation S541G was detected in strains AF1 and AF2. However, the effect of the combination of the *hmg1* mutation S541G with the *cyp51A* mutations TR34/L98H/S297T/F495I on azole resistance remains unclear.

Besides the mutation sites of genes associated with ergosterol synthesis, overexpression of these genes also plays an important role in azole resistance (Handelman et al., 2021). The results of this study also confirmed that the genes associated with ergosterol synthesis were mainly up-regulated in strains AF1, AF2, AF4, and AF8 after azole treatment (Figure 5). In addition, multidrug efflux pumps are composed of multilayer transmembrane structures, which play important roles in expulsion of drugs and various molecules from the cell (Rajendran et al., 2011). Overexpression of genes associated with efflux pump transporters has been linked to multidrug resistance in *A. fumigatus* (Costa et al., 2014). A previous study confirmed that genes associated with ABC drug efflux pumps (e.g., *atrF*, *cdr1B*, and *mdr1*) are related to azole resistance of *A. fumigatus* (Paul et al., 2018). Moreover, deletion of the MFS-related genes *mdrA* and *mfsB* significantly increased susceptibility to ITR and VRC (Meneau et al., 2016). In the present study, as compared to strain AF293, the ABC efflux pump-related gene *atrF* was down-regulated, while the MFS efflux pump-related gene *mdrA* was up-regulated and *mfsB* was down-regulated in strains AF1, AF2, AF4, and AF8 (Figure 6). In addition, azole treatment influenced the expression levels of genes associated with efflux pump transporters. These results suggest that azole resistance of the four strains is not only related to mutations and overexpression of genes associated with ergosterol synthesis, but also genes associated with efflux pumps.

**FIGURE 8:**
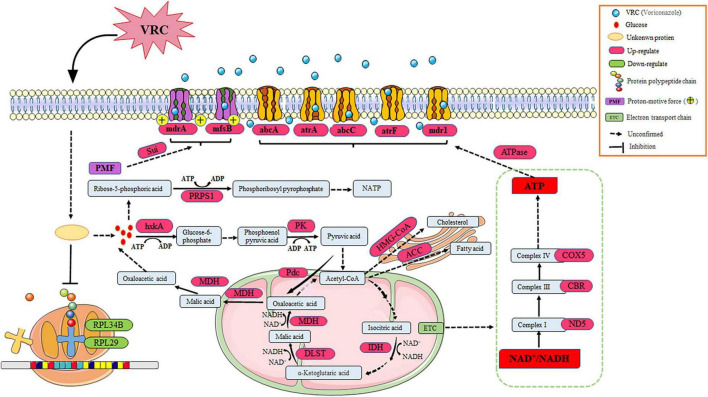
The comprehensive mechanism of resistance to VRC in AF5 strain. VRC reacts with unknown proteins to activate glycolysis, the tricarboxylic acid cycle, and the electron transport chain. Meanwhile, the ribosome metabolic pathway was inhibited, which prevented energy production for the ABC efflux pumps.

The molecular mechanisms of azole resistance in *A. fumigatus* primarily involve mutations in genes associated with ergosterol synthesis, upregulation of drug efflux pumps, and activation of cellular stress responses. Interestingly, *cyp51* of strain AF5 carried no mutation, indicating that *cyp51* does not participate in the mechanism of azole resistance of strain AF5. Furthermore, transcriptome sequencing found that after treatment of VRC, the expression of ribosome-related genes in AF5 strain was down-regulated, and the expression of drug-efflux pump related genes was up-regulated, which indicate that the non-*cyp51* resistance mechanism of AF5 strain was mainly related to activation of ABC efflux pumps, the glycolysis pathway, and the ribosome metabolic pathway. Our findings further validated that the application of VRC induces up-regulation of genes, associated with energy production pathways, down-regulation of genes, related to energy expenditure pathways, and an increase in the NAD^+^/NADH ratio in the AF5 strain (Figures 7G, H), these changes in the AF5 strain will reduce its own energy consumption and make the energy supply to the membrane transport pumps more adequate. It is known that ABC efflux pumps are mainly powered by energy from ATP (Biemans-Oldehinkel et al., 2006). Therefore, we postulated that under the pressure of VRC, AF5 strain can rapidly regulate intracellular energy supply and mobilize more energy to supply ABC efflux pumps, thereby facilitating drug efflux and reducing intracellular drug concentration. Based on these findings, the molecular mechanism of VRC resistance of strain AF5 is related to increased glycolysis, the tricarboxylic acid cycle, fatty acid and cholesterol synthesis, and inhibition of ribosomal protein synthesis to increase energy production for drug efflux pumps (Figure 8).

In summary, there were notable differences in the biological characteristics and pathogenic ability among the five test strains assessed in this study. The *cyp51A* mutations TR34/L98H/V242I/S297T/F495I combined with the *hmg1* mutation S541G were associated with the mechanism of ITR resistance of strains AF1 and AF2. In addition, the *cyp51A* mutations TR46/Y121F/V242I/T289A confer VRC resistance to strains AF4 and AF8. However, the VRC resistance mechanism of strain AF5 was related to changes in energy production and increased expression of genes associated with drug efflux pumps. This study provides reference for the discovery of a new mechanism of resistance to azoles by *A. fumigatus* and the development of drug targets.

## Data availability statement

The datasets presented in this study can be found in online repositories. The names of the repository/repositories and accession number(s) can be found in the article/Supplementary material.

## Ethics statement

The studies involving humans were approved by Clinical Trial Ethics Committee, Southwest Medical University. The studies were conducted in accordance with the local legislation and institutional requirements. Written informed consent for participation was not required from the participants or the participants’ legal guardians/next of kin in accordance with the national legislation and institutional requirements.

## Author contributions

ZS conceived and designed the research. FL, MZ, XZ, and CY conducted the experiments. YL and JZ contributed new reagents or analytical the tools. FL, CX, and GQ analyzed the data. MZ and ZS wrote the manuscript. All authors read and approved the manuscript.

## Footnotes

1
https://blast.ncbi.nlm.nih.gov/Blast.cgi

2
https://www.megasoftware.net/

3
www.biocloud.net
