# Supplementary material for: Comparative analysis of the biological characteristics and mechanisms of azole resistance of clinical Aspergillus fumigatus strains
Source: Front Microbiol. 2023 Nov 9;14:1253197. doi: 10.3389/fmicb.2023.1253197 (PMC10665732; doi:10.3389/fmicb.2023.1253197)

Comparative Analysis of the Biological Characteristics and Mechanisms of Azole Resistance of Clinical *Aspergillus fumigatus* Strains

Meng Zeng[1,2], Xue Zhou[1], Chunhong Yang[1], Yanfei Liu[3], Jinping Zhang[1], Caiyan Xin[1], Gang Qin[4], Fangyan Liu[1†*], Zhangyong Song[1†*]

[1]School of Basic Medical Sciences, Southwest Medical University, Luzhou 646000, China

[2]Department of Clinical Laboratory, Yongchuan Hospital of Chongqing Medical University, Chongqing 402160, China

[3]Department of Clinical Laboratory, The Affiliated Hospital of Qingdao University, Qingdao 266000, China

[4]Department of Otolaryngology Head and Neck Surgery, The Afliated Hospital of Southwest Medical University, Luzhou 646000, China

*** Correspondence:**

Corresponding Author Zhangyong Song* szy83529@163.com

Fangyan Liu1†*, Zhangyong Song1†* These authors are co-correspondence authors.

Author 1: Given name: Meng Family name: Zeng

Email: zengmeng19881026@163.com

Author 2: Given name: Xue Family name: Zhou

Email: zxlovebb0720@163.com

Author 3: Given name: Chunhong Family name: Yang

Email: yangyangya919@163.com

Author 4: Given name: Yanfei Family name: Liu

Email: 502194115@qq.com

Author 5: Given name: Jinping Family name: Zhang

Email: zhangjpwcx@163.com

Author 6: Given name: Caiyan Family name: Xin

Email: xincy0211@126.com

Author 7: Given name: Gang Family name: Qin

Email: qin-lzm@163.com

Author 8: Given name: Fangyan Family name: Liu

Email: liufangyan1989@163.com

Author 9: Given name: Zhangyong Family name: Song

Email: szy83529@163.com

Tel/Fax: +86-0830-3161506

Supplementary Table 1. Clinical information of patients with different test strains

Strain	Gender	Age (year)	Dasic disease	Diagnoses	Prognosis
AF1	Man	46	Acute lymphoblastic leukemia	Invasive pulmonary Aspergillosis	Survival
AF2	Man	49	Acute lymphoblastic Leukemia	Invasive pulmonary Aspergillosis	Survival
AF4	Woman	74	Anti-neutrophil cytoplasmic antibody associated vasculitis	Invasive pulmonary Aspergillosis	Death
AF5	Man	52	Anti-neutrophil cytoplasmic antibody associated vasculitis	Invasive pulmonary Aspergillosis	Death
AF8	Woman	73	Kidney transplant	Invasive pulmonary Aspergillosis	Survival

Supplementary Table 2 Primers were used in PCR amplification and RT-qPCR analysis

Accession number	Genes	Primer (5'→3')
Sequencing primers		
Afu4g06890	*cyp51A*	F:GTCTTTAGATTCGGTGGACGC<br>R:AATAAGGGTTCAATACAGTCAT
Afu7g03740	*Cyp51B*	F:GCGAGACACTCAGCCTACCTTTA<br>R:GACGGCAGAATACCCAGAAGTGATG
NR_121481	*ITS*	1:TCCGTAGGTGAACCTGCGG<br>4:TCCTCCGCTTATTGATATGC
Afu2g03700	*hmg1*	F:GTGTCTCACCCCTGCTAACCTTCAA<br>R:AGGCTCGGCTACATTCCTCTCTTGG
RT-qPCR primers		
Afu6g04360	*atrF*	F:AGTCCAGCTACACGGTTTCA<br>R:TGTCGAGGGATTCACCGTAG
Afu1g14330	*cdr1B*	F:GCCTCGACAGTCAAACTTCC<br>R:GTCGAATCGTTGGAACAGCA
Afu4g06890	*cyp51A*	F:TCCAAGCTGATGGAGCAGAA<br>R:GTGAATCGCGCAGATAGTCC
Afu7g03740	*cyp51B*	F:GCGGGTCAACATTCTTCCTC<br>R:GAGGCAAGTCAGATCCGAGA
Afu5g07780	*erg1*	F:CAAGCAACATCACCCGCATA<br>R:TTCGGGAATGTCAACGAGGA
Afu5g14350	*erg4*	F:CATGTGACGTGTTCTTCGCT<br>R:ACCCGACGCTCATATTCCAT
Afu1g03150	*erg24*	F:TTTAGCCCTGGGCACATACA<br>R:CTGGTGCCAATTCTCTCAGC
Afu2g03700	*hmg1*	F:ACCACCATCCTCTGCATCAA<br>R:ATTCGGTCAGAGGCCAATCA
Afu5g06070	*mdr1*	F:CCCTGAGCTTGGTTACATGC<br>R:GCTAATTGCCTTGGCGTACA
Afu4g10000	*mdr2*	F:ATTGGAGCCAAAGTCGAACG<br>R:CTCGATGTCCTCATGCTCCT
Afu1g12690	*mdr4*	F:GGTATCGTCTTCGGTGACCT<br>R:CACTCACCAAACACCAGCAA
Afu1g13800	*mdrA*	F:GTCCAAGTTCAAGCCAAGCA<br>R:GACGGGTAATTGGTCCAAGC
Afu1g15490	*mfsB*	F:TACCTGGCACTCATCAGCAA<br>R:TCGAACCGAGAACCTTGGAA
Afu2g08670	*ACC*	F:GCTCGGTGGTACTCAGATCA<br>R:TTCTTGTCGGGAACGAAGGA

Afu5g05520	*ATPase*	R:TCACGACTCAAGGGTCACAA<br>F:ATTGTCCACGCTATGGCAAC
Afu1g04540	*CBR*	R:GGCCAACAAGCACAAGCATA<br>F:GAGAATGTCCTCCTCACGGA
Afu5g10560	*COX5*	R:GAACTGTGGATGCAGCTGAG<br>F:GTGAGGACCGAAAGCAATCC
Afu3g05370	*DLST*	F:TCAGATGGCGGAATCGATCA<br>R:CCAACCGTCACAGTGTCTTC
Afu7g04500	*His1*	F:GATACCGTCGTCGAAAGCAC<br>R:AGCGGTGATGACACCACTAA
Afu2g03700	*HMG-CoA*	F:AGAGCCAGATGATGGAGACG<br>R:CGATATCGAAAGTGCGGCTT
Afu1g12800	*IDH*	F:CAACCTGTTTGCGAACTTGC<br>R:TCTGGACAACACCATCGACA
Afu2g13800	*MDH*	F:TCCTGCCTTGCCTCACATAA<br>R:GACTGAGGAGCATGGCAAAG
Afu6g00750	*Pdc*	F:GCTGTCCATCGGATACATGC<br>R:TCAGCTCTTGGACGGTCATT
Afu1g06960	*PDHA1*	F:TCGGTCAGGAAGCTGTTTCT<br>R:CCTTTCCGTAGGCGATACCT
Afu6g07430	*PK*	F:CCGGCAAGCTGATCTATGTG<br>R:GGGAAGATCAACGTCAGTGC
Afu3g13380	*PRPS1*	F:GCCTTCGATCCACTGTTGTC<br>R:GAGCCACCCATTGCTTGTAG
Afu2g02275	*Sui*	F:GTGTCGTCATCCTCTACGGA<br>R:GTGCTTGACGTTCTTAGCGT
Afu1g03110	*RPL29*	F:ATGGCCAAGTCCAAGAACG<br>R:CTTGAGGGAAGGGTAACGGT
Afu2g03040	*RPL34B*	F:TTTCTTGGTGATGGGCAAGC<br>R:TTGTCGTCGTTGTCCTCGTA
Afua1g10910	*β-tubulin*	F:TTCCCAACAACATCCAGACC<br>R:CGACGGAACATAGCAGTGAA

Note: F: Forward; R: Reverse

Supplementary Figure 1. Melanization of the larva of *Galleria mellonella* 24 hours after infection with *Aspergillus fumigatus* conidium. UTC: blank control group, PC: puncture without injection group, PBS: PBS injection group.

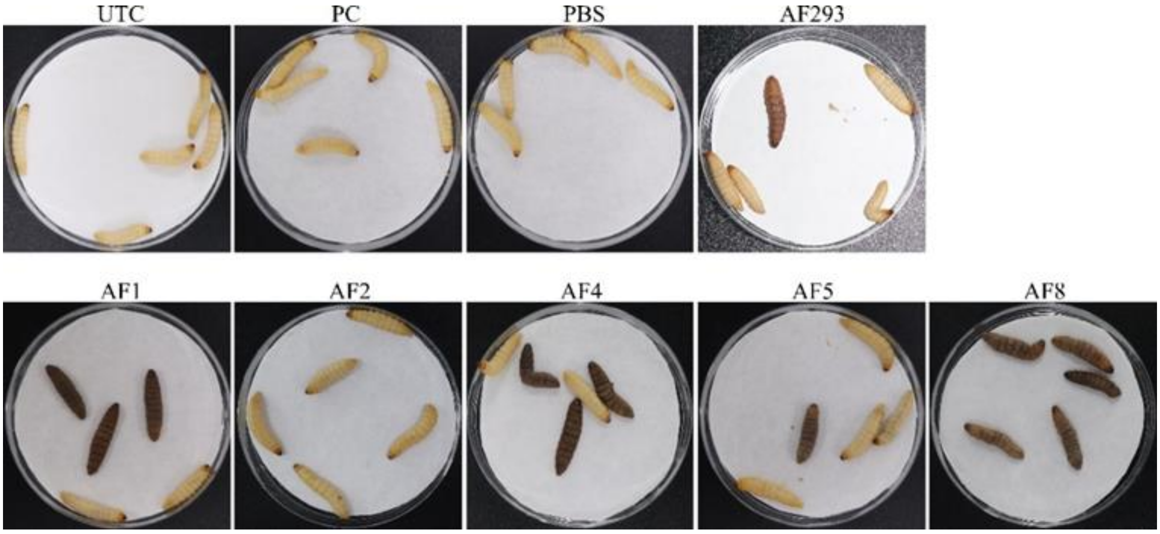

Supplementary Figure 2. Susceptibility to various antifungal drugs was determined by the e-test method. photos were taken after culture at 37℃ for 24 h. ANI, anidulafungin; AMB, amphotericin B; CAS, caspofungin; ITR, itraconazole; ISA, isavuconazole; MF, micafungin; VRC, voriconazole.

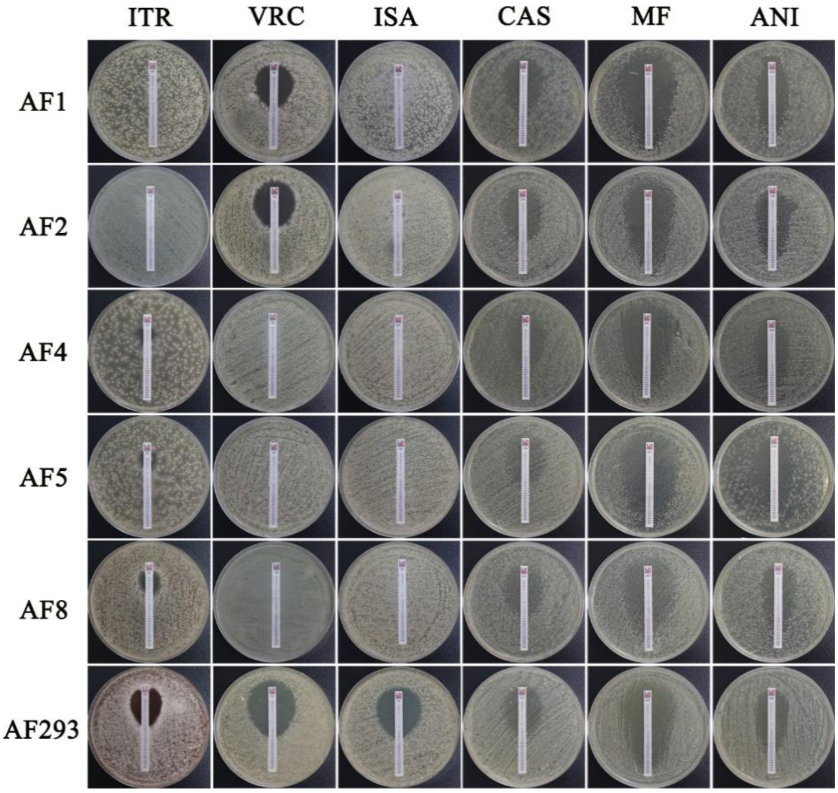

Supplementary Figure 3. The 3D structures of the Cyp51A proteins of the test strains.

Strains are shown in blue and AF293 strain is shown in red.

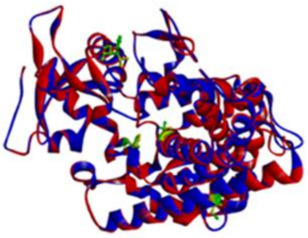

AF293-AF1

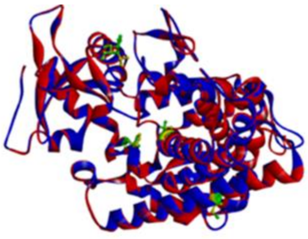

AF293-AF2

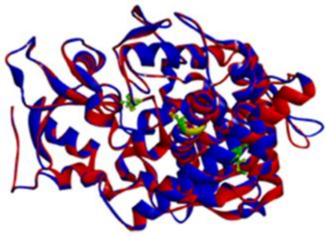

AF293-AF4

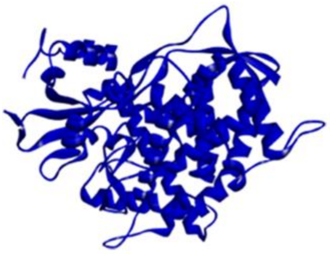

AF293-AF5

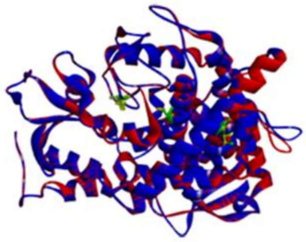

AF293-AF8

Supplementary Figure 4. Analysis of functional categories of DEGs in AF5 strains.

A

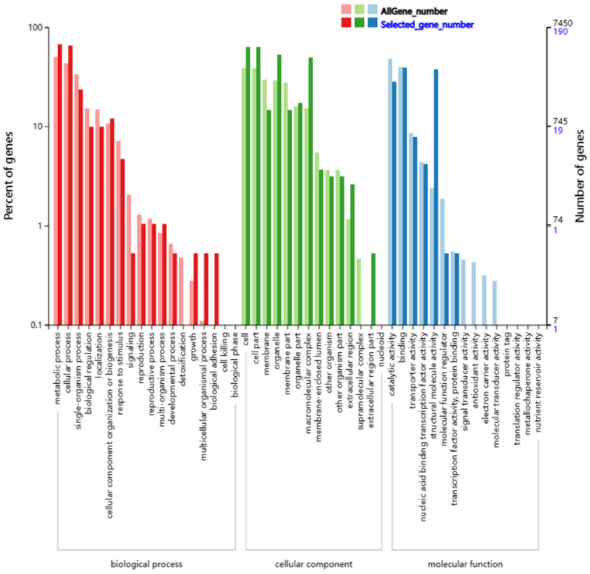

Supplementary Figure 5. Analysis of GO biological process of DEGs in AF5 strain.

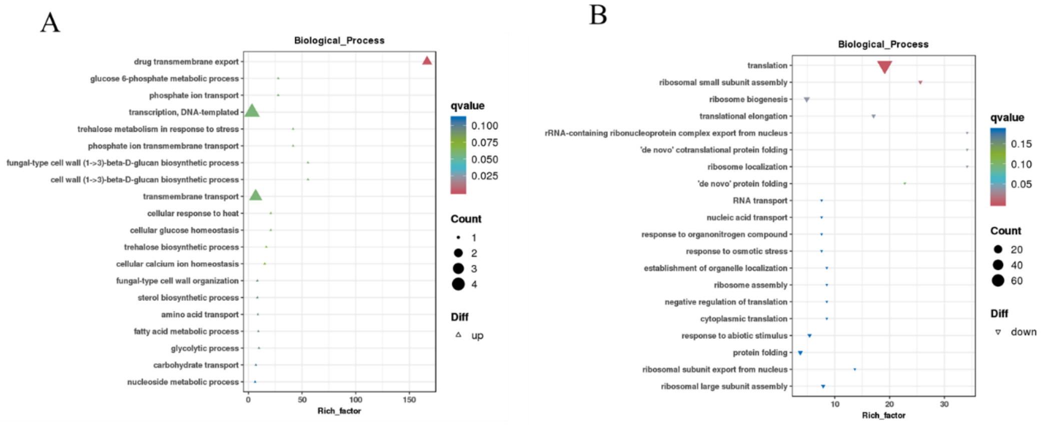

Supplement: Supplementary file 1 [file Presentation_1.pdf]
